# Locomotion Inhibition of *Cimex lectularius* L. Following Topical, Sublethal Dose Application of the Chitin Synthesis Inhibitor Lufenuron

**DOI:** 10.3390/insects8030094

**Published:** 2017-09-01

**Authors:** Brittany Campbell, Rebecca Baldwin, Philip Koehler

**Affiliations:** Department of Entomology and Nematology, University of Florida, Gainesville, FL 32611, USA; baldwinr@ufl.edu (R.B.); pgk@ufl.edu (P.K.)

**Keywords:** bed bug, chitin synthesis inhibitor, effective dose, pulling force, topical

## Abstract

To date, few studies have evaluated chitin synthesis inhibitors against bed bugs, although they would provide an alternative mode of action to circumvent insecticide resistance. Acute and sublethal effects of lufenuron were evaluated against two strains of the common bed bug. Combined acute and sublethal effects were used to calculate effective doses. The dose that was effective against 50% of Harlan strain bed bugs was 0.0081% (*w*/*v*), and was much higher against Bradenton strain bed bugs (1.11% *w*/*v*). Sublethal doses were chosen to determine the effect that leg abnormalities had on pulling force. Both Harlan and Bradenton strain bed bugs had significantly lower locomotion ability (*p* < 0.0001) following topical application of lufenuron. The observed sublethal effects that limit locomotion could prevent bed bugs from moving within a domicile and taking a blood meal, subsequently reducing a bed bug population over time.

## 1. Introduction

Liquid chemical insecticide applications are advantageous for bed bug control because of their low cost and ease of application as compared to other control methods (e.g., heat and fumigation). Pest control companies in the United States rely heavily on pyrethroid insecticide applications for bed bug treatments [1]. Furthermore, the majority of pesticides labeled for indoor use in the United States contain pyrethroids as the active ingredient; consequentially, bed bugs have been frequently exposed to these insecticides.

The frequent exposure of common bed bugs (*Cimex lectularius*) to pyrethroids has resulted in significant resistance to these active ingredients [2,3,4,5,6,7]. Resistance has also developed in bed bugs to neonicotinoid insecticides that are often combined and formulated with a pyrethroid [8]. However, rotating and utilizing insecticides with different modes of action, as well as other integrated pest management strategies, can circumvent insecticide resistance problems. Unfortunately, there are limited products available with alternative modes of action to pyrethroids available for a bed bug insecticide rotation program.

Insect growth regulators (IGRs) have an alternative mode of action to pyrethroid insecticides; affecting insect growth, development, and reproduction. Insect growth regulators have been found to be highly effective against multiple urban insect pests, including flies [9,10,11,12,13], fleas [14,15,16,17], termites [18,19,20,21], and cockroaches [22,23,24,25]. However, their use has not been extensively investigated in bed bugs. There is currently only one insect growth regulator that is labeled for bed bug control in the United States, manufactured under the trade name Gentrol ((S)-hydroprene; Wellmark International; Schaumberg, IL, USA).

(S)-Hydroprene is a juvenile hormone analog that affects multiple developmental processes (i.e., ecdysis and formation of reproductive organs) in insects that are regulated naturally by the presence of juvenile hormone during ecdysis. Although Gentrol is a registered insecticide in the United States for bed bug control, Gentrol^®^ aerosol and Gentrol^®^ concentrate ((S)-hydroprene; Wellmark International; Schaumberg, IL, USA) require application rates ≥ 3× the label rate to achieve 66–100% adult bed bug mortality [26]. Similar to Todd (2006) [26], (S)-hydroprene was not effective against bed bugs except at elevated label rates. Applications at 10× the label rate caused a 100% reduction in bed bug oviposition in one bed bug strain, but only a 38% ovipositional reduction in another strain [27].

The addition of insect growth regulators to an integrated pest management program (IPM) for bed bugs has potential because IGRs exhibit low mammalian toxicity [27], which would be advantageous for indoor use, as well as providing an alternative mode of action for rotation in a chemical program. The limited studies available on bed bugs and IGRs have mostly investigated juvenile hormone analogs (JHAs), and have largely neglected another type of IGR, the chitin synthesis inhibitors (CSIs). This may be primarily because CSIs are known to be highly effective when ingested (i.e., as baits or foliar treatments), but are not often used as contact insecticides [28].

Chitin synthesis inhibitors impede the biosynthesis of chitin. As a result, the cuticle is usually malformed following ecdysis, causing morphological abnormalities or death. Few studies have investigated the efficacy of chitin synthesis inhibitors against bed bugs, and there are currently no insecticides labeled in the United States as containing a chitin synthesis inhibitor for bed bug control. However, one insecticide, under the trade name Tenopa (BASF, Ludwigshafen, Germany) is registered in Europe, South America, and Mexico, and contains a pyrethroid (alpha-cypermethrin) and a chitin synthesis inhibitor (flufenoxuron). Flufenoxuron has been evaluated against first and second instar bed bugs previously [29]. Flufenoxuron caused morphological abnormalities and approximately 82% mortality 35 days after treatment to insecticide impregnated filter papers.

The purpose of this study was to evaluate the lethal and sub lethal effects of the chitin synthesis inhibitor lufenuron on bed bugs. Fifth instar bed bug ecdysis, morphological abnormalities, and mortality was evaluated following the topical application of lufenuron to individual bed bugs.

The resultant bed bug adults that molted with leg malformations after sublethal exposure were used to quantify the effects of malformations on locomotion ability. Locomotion ability was measured using pulling force assays to determine the force bed bugs generated when they attached their tarsae to a surface. Pulling force assays have been used previously to evaluate a bed bugs ability to climb different textured surfaces [30], as well as to measure the ability for tropical bed bugs, *Cimex hemipterus*, to generate vertical friction and escape pitfall traps used for bed bug monitoring [31].

## 2. Materials and Methods 

### 2.1. Insects

Two strains of bed bugs (Harlan and Bradenton) were used for the topical application and pulling force assays. The Harlan strain was collected in 1973 in Fort Dix, NJ, USA, and then was maintained in a laboratory on human blood. Our laboratory acquired this strain in the late 2000s. The Bradenton strain was collected by a pest control company in Bradenton, FL in August 2013.

Bed bugs were fed weekly on defibrinated rabbit blood (Hemostat, Dixon, CA, USA) using an artificial feeding system [32], and maintained at approx. 70% RH, 25 °C, and a 12:12 L:D photoperiod. All bed bug colonies were maintained in plastic jars (Mold-Rite Plastics, Plattsburg, NY, USA, 300-mL) enclosed at one end with mesh for feeding, with accordion-style folded filter paper (diam. = 9 cm, # 2, Whatman, GE Healthcare UK limited, Buckinghamshire, UK) provided for harborage.

### 2.2. Insecticide Dilutions

Technical grade lufenuron (FMC Corporation, Philadelphia, PA, USA) was weighed on an analytical balance and then serially diluted 10-fold for Harlan strain bed bugs and 2-fold for Bradenton strain bed bugs with acetone into five concentrations. A control treatment consisted of only acetone. The serially diluted concentrations of lufenuron for topical application to Harlan strain bed bugs were 0.000016, 0.00016, 0.0016, 0.016, and 0.16% (*w*/*v*). Topical applications of lufenuron to Bradenton strain bed bugs were diluted to concentrations of 0.32, 0.64, 1.28, 2.56, and 5.1% (*w*/*v*). The entire experiment was replicated three times.

### 2.3. Insect Growth Regulator Topical Application Bioassay

Individual bed bugs were topically treated with five different doses of 1 µL of technical grade lufenuron to the ventral side of their abdomen using a Hamilton syringe mounted on a repeating dispenser (50 µL; Hamilton Company, Reno, NV, USA). The bed bugs were placed inside of an aluminum weigh dish (6.4 cm dia., 1.7 cm ht., Fisher Scientific, Waltham, MA, USA) chilled on ice to restrict movement during the application of lufenuron. One dose consisted of 15 bed bugs individually treated with lufenuron that were then placed in a cohort of 5 bed bugs for feeding, thus resulting in three replicates/dose. An entire experiment consisted of five doses and a control treatment for a total of 90 treated bed bugs.

Fifth instar bed bugs were fed in a cohort for ease of feeding on rabbit blood (Hemostat, Dixon, CA, USA) 1 day after topical application of lufenuron. For feeding, bed bug cohorts were placed into a glass vial (20 mL, polypropylene caps, Wheaton, Millville, NJ, USA) and the vial was enclosed with mesh (90 µm, nylon, Amazon supply, Seattle, WA, USA) on the open end. The mesh was then covered with parafilm (“M”, 10.16 cm width, Bemis, Neenah, WI, USA) and the vial was inverted directly onto defibrinated rabbit blood that was held in a soufflé cup (30 mL, DART, Mason, MI, USA) placed on the top of a hot plate (Isotemp, Fisher Scientific, Waltham, MA, USA) maintained at ~40 °C to simulate human body temperature. This method differed from the methodology of colony maintenance, to limit the amount of blood wasted for feeding small cohorts of bed bugs. Furthermore, this feeding method encouraged bed bugs to feed more quickly, since they were placed directly on top of the blood.

Following the topical application of the insecticide, the bed bugs were placed into a Petri dish (Polystyrene, 6.0 × 1.5 cm; Fisher Scientific, Waltham, MA, USA) containing filter paper (# 1, 4.3 cm diam.; Whatman, GE Healthcare UK limited, Buckinghamshire, UK). Bed bugs that did not feed to repletion following topical application of lufenuron were excluded from further analysis (<10%). Bed bug mortality, morbidity, and molting were recorded 14 days after treatment. Mortality was recorded as those insects that did not move when probed. Morbidity was recorded as insects that were still alive but exhibited restricted movement due to morphological deformities and had an extreme reduction in responsiveness following prodding. Further analysis of leg abnormalities and locomotion inhibition was quantified with pulling force assays using bed bugs exposed to lufenuron that did not result in high mortality, but high levels of morphological abnormalities.

### 2.4. Locomotion Inhibition Quantified Using a Pulling Force Assay

A dose that resulted in an approximate dose that effected 25% of the population (ED_25_) was used to evaluate the effects of lufenuron on locomotion inhibition. The dose selected for Harlan strain bed bugs was 0.0016% (*w*/*v*), and 0.64% (*w*/*v*) lufenuron for Bradenton strain bed bugs. Twenty bed bugs were individually measured from control (acetone-only) treatments and the doses previously mentioned per strain for a total of 80 bed bugs.

**Figure 1:**
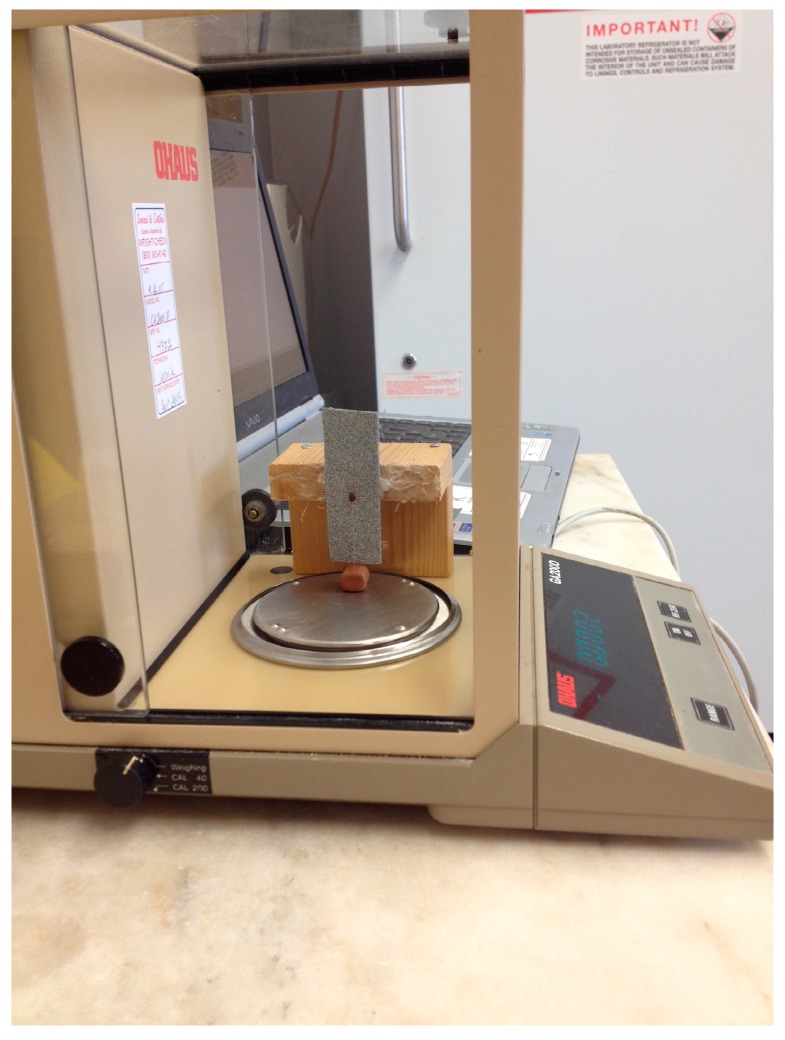
Photograph of a pulling force assay on an analytical balance. Here, a bed bug is pictured gripping the rough sandpaper surface on the wooden platform.

Methods similar to [30] were used to calculate the pulling force of bed bugs that were either exposed to lufenuron or to acetone alone (control). Briefly, individual bed bugs were tethered to a paint brush bristle using super glue (Loctite; Henkel Corporation, Rocky Hill, CT, USA) attached at the first or second segment of the dorsal abdomen. Sandpaper (Aluminum Oxide, 60 grit; 3M, St. Paul, MN, USA) was mounted to a glass microscope slide (Premium microscope slides plain; Fisher Scientific, Pittsburgh, PA, USA) and then the slide with the sandpaper attached was mounted to a wooden platform. The end of the paintbrush bristle (4 cm long, polyester; Great American Marketing, Valencia, CA, USA) that was not attached to the bed bug and loose was inserted into a ball of modeling clay (2.53 g; Van Aken International, Charleston, SC, USA). The wooden platform with the attached sandpaper surface was placed outside of the weighing pan within an analytical balance (New Classic MF, Model MS105D4; Mettler Toledo, Grietensee, Switzerland) and the tethered bed bug in the clay ball was placed directly onto the analytical balance. After the balance was tared to zero, the wooden platform with the sandpaper surface attached was moved forward, without directly contacting the weighing pan, until the bed bug could grip the surface (Figure 1).

Once all of the six bed bug tarsi contacted the sandpaper surface, negative mass changes (indicative of the mass pulled by the bed bug) were recorded directly from the analytical balance software for 240 s. The mass data was then converted to force using the formula F = ma (F = force [mN], m = mass (g), and a = acceleration (m^2^/s [acceleration due to gravity was a constant −9.81 m^2^/s]). The maximum amount of force and the average amount of force (mean of several readings over 240 s) generated by each bed bug was then calculated after the five-minute duration. 

### 2.5. Statistical Analysis

Insecticide doses were chosen that resulted in mortality and malformations (leg abnormalities, cuticle abnormalities that reduced bed bug responsiveness) ranging from 10–80%. Effective doses (ED) were chosen instead of lethal doses because mortality did not reach 80%; however, significant morphological effects were observed that limited bed bug movement and responsiveness (recorded as malformed). The ED_50_ was calculated using a generalized linear model with a binomial distribution and probit link using JMP (JMP Pro 13; SAS institute, Cary, NC, USA). The maximum amount of force and average amount of force generated between the bed bugs exposed and not exposed to the ED_25_ doses of lufenuron were evaluated using t-tests in JMP for both Harlan and Bradenton strains. Values of *p* ≤ 0.05 were used to indicate significance.

## 3. Results

The effective dose that resulted in 50% malformations and mortality (ED_50_) for Harlan strain bed bugs was 0.0081 (% *w*/*v*) [95% CI = 0.0021−0.014] lufenuron. The ED_50_ of lufenuron for Bradenton strain bed bugs was much higher compared to Harlan strain bed bugs, with a value of 1.11 (% *w*/*v*) [95% CI = 1.10−1.22]. Mortality from lufenuron did not result in a typical dose response, which is unlike characteristic responses of insects to neurotoxins (Table 1). However, in general, mortality increased to some extent with an increase in the dose applied (Table 1). As the dose of lufenuron increased, the observed effects transitioned from sublethal to lethal (Table 1).

**Figure 2:**
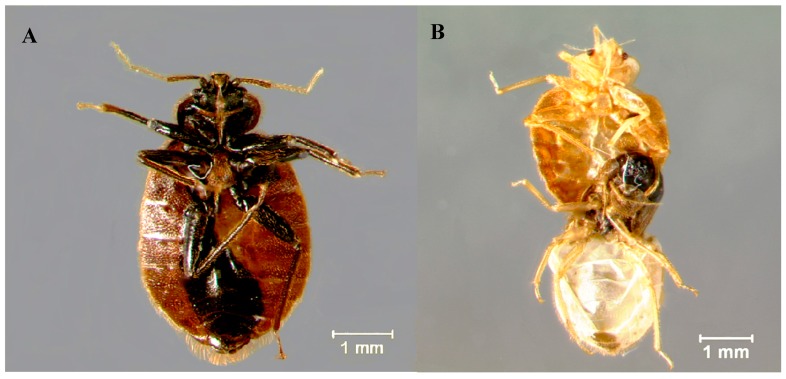

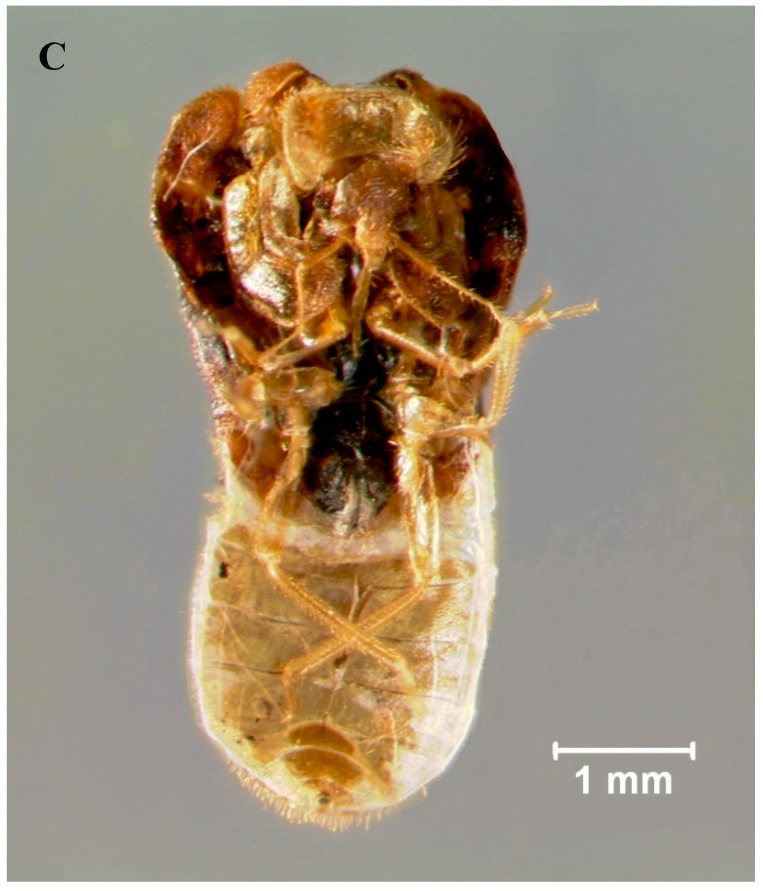
Photographs showing the lethal effects of lufenuron following ecdysis of treated 5th instar Harlan bed bugs with a dose of 16% (*w*/*v*) lufenuron. (**A**) A fully molted adult bed bug that died shortly after emerging from the exuvia; (**B**) A fifth instar that died during the process of molting with an extrusion of internal stomach structures; (**C**) A fifth instar bed bug that died during the molting process and could not fully emerge from its exoskeleton.

**Figure 3:**
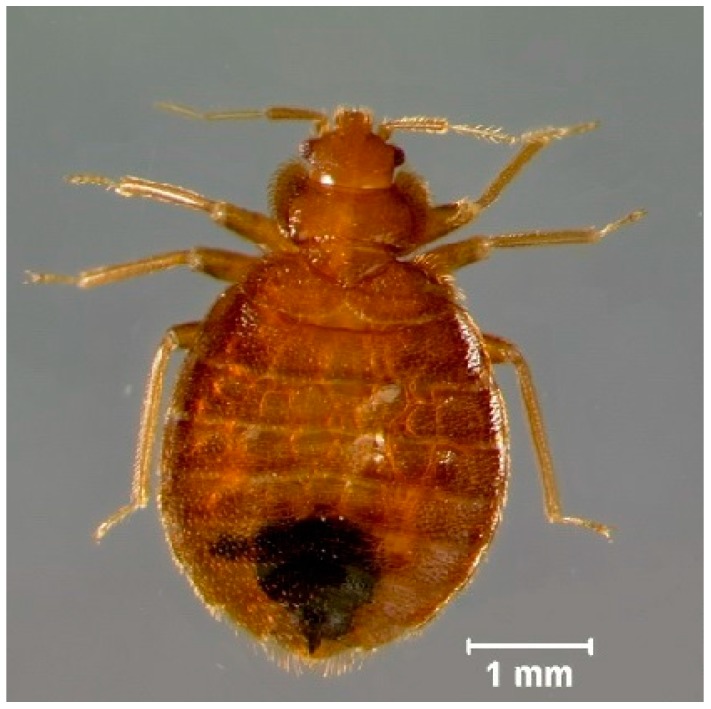
Sublethal effect following topical application of 0.0016% (*w*/*v*) lufenuron on a Harlan strain bed bug. Complete ecdysis occurred; however, the bed bug could not properly walk and could not fold its legs underneath its body.

**Figure 4:**
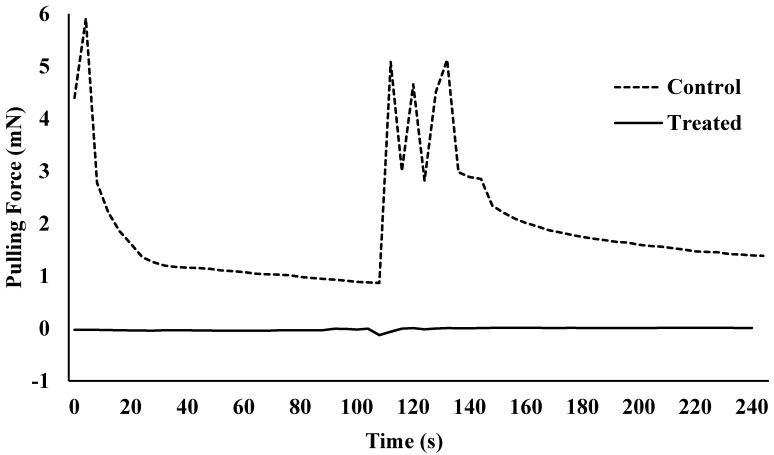
Pulling force over time for one Harlan strain bed bug that was non-treated (control) or treated with lufenuron (0.0016% *w*/*v*).

Lufenuron caused multiple morphological problems (Figure 2 and Figure 3) that resulted in the decreased locomotion of bed bugs following the molt from 5th instar to adult. Lufenuron had a significant effect on bed bug locomotion following ecdysis for both Harlan strain and Bradenton strain bed bugs. Most Harlan strain bed bugs that were treated could not generate any pulling force, as compared to one representative non-treated bed bug that was able to grip and pull on the sandpaper surface at a maximum of 6 mN (Figure 4).

**Figure 5:**
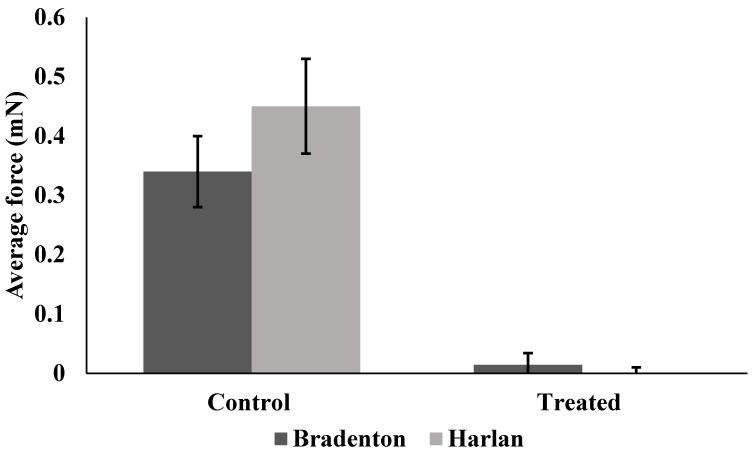
Average amount of force generated by Bradenton and Harlan strain bed bugs when gripping a surface with tarsi following no exposure to lufenuron (Control) or exposure to sub-lethal doses of lufenuron (Treated).

**Figure 6:**
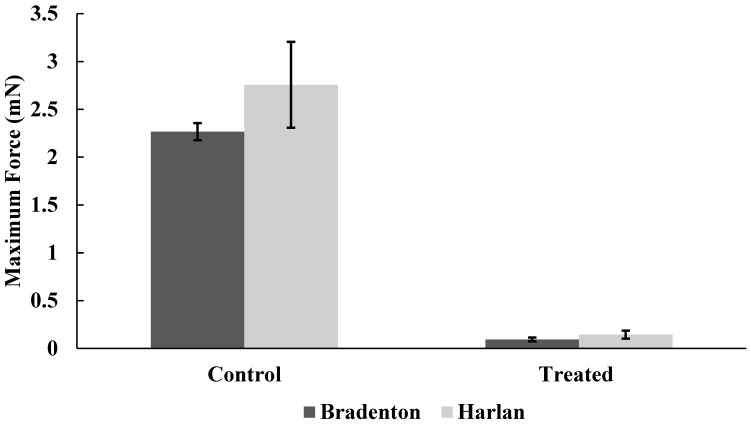
Maximum amount of force generated by Bradenton and Harlan strain bed bugs when gripping a surface with tarsi following no exposure to lufenuron (Control) or exposure to sub-lethal doses of lufenuron (Treated).

Harlan strain bed bugs treated with lufenuron were significantly less able to grip the sandpaper surface, as indicated by the reduction in the average force when compared to non-treated bed bugs (f = 5.35, df = 22, *p* < 0.0001) (Figure 5), as well as the maximum amount of force generated across all readings over time (f = 6.8, df = 32.75, *p* < 0.0001) (Figure 6). Bradenton bed bugs treated with lufenuron also had a significant reduction in the average amount of force they could generate to grip onto a surface (f = 8.86, df = 23.97, *p* < 0.0001) (Figure 5) as well as the maximum amount of force generated (f = 12.03, df = 30.80, *p* < 0.0001) (Figure 6).

## 4. Discussion

The benzoylurea compounds have been documented to cause multiple effects directly related to chitin synthesis, however the mode of action of CSIs has not been entirely determined [33]. Studies have suggested that CSIs inhibit the action of chitin synthase, which is an integral protein that aids in the synthesis of *N*-acetylglucosamine [34]. Conversely, the mode of action of the CSI diflubenzuron has been suggested to inhibit the incorporation of *N*-acetylglucosamine into insect chitin during the molting process [35]. Nevertheless, the external physiological ramifications of chitin synthesis inhibitors have been observed and reported in numerous insect taxa. Chitin synthesis inhibitors impede insect ecdysis, often resulting in malformations in the newly formed cuticle of an insect and can also affect the peritrophic matrix and intestinal system [33].

Previous studies have documented that chitin synthesis inhibitors have a broad range of efficacy against numerous insect pests, and they also interfere with hemipteran ecdysis. For example, the chitin synthesis inhibitor diflubenzuron caused the incomplete ecdysis of last instar milkweed bugs, *Oncopeltus fasciatus* Dallas, when topically applied at the penultimate life stage [36]. The predatory bug, *Podisus maculiventris* say, was not able to molt from the penultimate stage to adult after feeding on insects dipped in label rates for field application of the chitin synthesis inhibitor novaluron [37].

The chitin synthesis inhibitor lufenuron had a significant effect on the ecdysis of fifth instar bed bugs to adult. Lufenuron caused mortality during, or immediately following ecdysis, resulting in insects with extreme cuticular deformities. Bed bugs that died following treatment had multiple abnormalities associated with chitin biosynthesis inhibition. For instance, some bed bugs did not fully emerge from the previous exuvia during ecdysis, or their intestines ruptured within the cuticle causing hemolymph to spread to their extremities, or their intestines penetrated externally through the newly formed cuticle, causing death.

Higher doses of lufenuron were required for efficacy against Bradenton strain bed bugs as compared to the Harlan strain that had been maintained in a lab for >30 years. This strain has exhibited levels of resistance to pyrethroid insecticides previously [38]; however, chitin synthesis inhibitors have an entirely different mode of action, acting on chitin synthesis rather than the nervous system. Therefore, we hypothesize this strain may have some cuticular resistance which has been demonstrated in other bed bug strains [7] that would also confer resistance from topical absorption of other insecticide types, including chitin synthesis inhibitors.

Most insecticidal efficacy studies report survival and mortality data, although sublethal effects may be equally as important in controlling or reducing a pest population [39]. Sublethal doses of lufenuron to fifth instar bed bugs resulted in significant issues with cuticular integrity and structure, consequentially causing leg malformations. Sublethal exposure of the chitin synthesis inhibitor novaluron to the Colorado potato beetle, *Leptinotarsa decemlineata*, resulted in beetles with poor walking ability and caused them to fall off of plants [40].

Bed bugs exposed to sublethal doses of lufenuron in our study held their legs extended from their bodies and demonstrated a limited walking ability (i.e., could not hold their body upright to walk, could not walk at all, or walked extremely slowly). Their ability to grip a rough surface was almost entirely impeded, exemplified by loss of generated force by treated bed bugs in the pulling force assays. Bed bugs that encountered smooth surfaces with no insecticide application were not very successful at gripping those surfaces [30] and, undoubtedly, bed bugs treated with a sublethal dose of lufenuron would not be able to navigate smooth surfaces. Alternatively, we tested the pulling force of bed bugs on a rough sandpaper surface, and the treated bed bugs could not grip that surface and generated a minute amount of force. Therefore, in almost any environment with a multitude of surfaces, bed bugs affected by sublethal doses of lufenuron would not be mobile enough to navigate the environment and reach a host for a blood meal.

## 5. Conclusions

The documented widespread resistance to pyrethroid insecticides and the recently discovered resistance to neonicotinoids limits the effectiveness of products available for bed bug control. Juvenoids are currently used for bed bug control; however, the limited research available on these products suggests that the label rate of the one product currently used for bed bug control in the United States has limited efficacy at the currently suggest label rate. Therefore, chitin synthesis inhibitors would be a novel insecticide for rotational use in a bed bug integrated pest management program.

## Figures and Tables

**Table 1:** Malformations and mortality of Harlan and Bradenton strain bed bugs following topical application of lufenuron for each tested dose.

Strain	Dose (% *w*/*v*)	n	# Dead	# Malformed	# Affected ^1^	% Affected ^2^	% Malformed/Affected ^3^
Harlan							
	0.000016	55	4	0	4	7	0
	0.00016	55	1	0	1	2	0
	0.0016	55	4	33	37	67	89
	0.016	55	20	18	38	69	47
	0.16	85	36	16	52	61	31
Bradenton							
	0.32	55	8	18	26	47	69
	0.63	55	21	19	40	73	48
	1.25	55	28	15	43	78	35
	2.5	55	17	12	29	53	42
	5.0	85	28	10	38	45	26

^1^ # Affected = (# dead + # malformed); ^2^ % Affected = (# affected /n)*100; ^3^ % Malformed/Affected = (# malformed/# affected)*100.
